# Terminal Decline in Physical Function in Older Adults

**DOI:** 10.1093/gerona/glad119

**Published:** 2023-05-06

**Authors:** Erwin Stolz, Hannes Mayerl, Graciela Muniz-Terrera, Thomas M Gill

**Affiliations:** Institute of Social Medicine and Epidemiology, Medical University of Graz, Graz, Austria; Institute of Social Medicine and Epidemiology, Medical University of Graz, Graz, Austria; Centre for Clinical Brain Sciences, University of Edinburgh, Edinburgh, UK; Heritage College of Osteopathic Medicine, Ohio University, Athens, Ohio, USA; Department of Internal Medicine, Yale School of Medicine, New Haven, Connecticut, USA

**Keywords:** Change points, End of life, Gait speed, Motor decline, Mortality

## Abstract

**Background:**

It is currently unclear whether (and when) physical function exhibits a terminal decline phase, that is, a substantial acceleration of decline in the very last years before death.

**Methods:**

702 deceased adults aged 70 years and older from the Yale PEP Study provided 4 133 measurements of physical function (Short Physical Performance Battery, SPPB) up to 20 years before death. In addition, continuous gait and chair rise subtest scores (in seconds) were assessed. Generalized mixed regression models with random change points were used to estimate the onset and the steepness of terminal decline in physical function.

**Results:**

Decline accelerated in the last years of life in all 3 measures of physical function. The onset of terminal decline occurred 1 year before death for the SPPB, and at 2.5 and 2.6 years before death for chair rise and gait speed test scores, respectively. Terminal declines in physical function were 6–8 times steeper than pre-terminal declines. Relative to those whose condition leading to death was frailty, participants who died from dementia and cancer had an up to 6 months earlier and 3 months later onset of terminal decline in SPPB, respectively.

**Conclusions:**

Terminal decline in physical function among older adults is comparable to the more established terminal decline phenomenon in cognition. Our results provide additional evidence of late-life rapid decline in physical function due to impending death.

Physical function declines during old age (1–4), and low levels of physical performance in mobility, balance, and muscle strength are associated with increased mortality (5–7). According to the terminal decline (TD) hypothesis (8,9), declines in the final years of life are not only due to aging but also, in part, due to mortality-related processes, that is, when the underlying disease(s) that cause(s) death advance significantly. More specifically, the TD hypothesis suggests that late-life decline is characterized by 2 distinct stages: a long pre-terminal phase of stability or only minor age-related decline followed by a shorter terminal phase of steep decline that ends with death. From its very beginning (10,11), research on TD focused on cognition and neglected the domain of physical function (9,12). A body of research shows that TD in global cognitive functioning is common and sets in on average between 3 and 7 years before death although the estimates vary by cognitive function examined (13). In contrast, it is still unclear whether (and when) older adults’ physical function also exhibits a phase of TD during the final years of life.

There is indirect evidence for TD in the domain of physical function as studies show that (a) declines in physical function are associated with increased mortality (2,14–17), (b) physical performance trajectories are steeper according to time-to-death than according to chronological age (18–20), and (c) that declines in performance measures are steeper among decedents than survivors (17,21). Direct evidence has been difficult to obtain, as TD describes a nonlinear, within-person change in the level of functioning whose estimation requires extensive longitudinal performance data from deceased older adults, ideally from cohorts with near-complete mortality. Such data with multiple repeated physical performance measurements covering the last 10 years of life (or more) (13) and including observations in close proximity to death are still rare (3), and even when available, subsequent estimation of the associated change point models remains computationally challenging (22). To our knowledge, TD in physical function has been directly assessed only in a single previous study. Specifically, Wilson and colleagues (23) analyzed composite scores of multiple physical performance measures in a small sample (*n* = 124) of older nuns and priests with 7 or more annual observations using mixed regression models with random change points. In this study, the authors found that TD in global motor function set in on average 2.5 years before death, that there was considerable between-person heterogeneity in the onset of TD (±1.3), and that onset of TD in physical functioning correlated closely with the onset of TD in cognitive functioning.

Against this backdrop, it is clear that more evidence is needed to establish whether and when there is TD in physical function. Knowledge about the existence and the timing of the onset of physical TD might help to identify which performance measure(s) can act as early markers of subsequent decline, and whether patterns of TD vary across subpopulations. For example, a later and steeper TD in the rate of health deficit accumulation has been reported for men compared to women (24), different TD trajectories in weight loss have been found according to age at death (25), and there is evidence that end-of-life trajectories of disability are modulated by the underlying cause of death (25–27). Identifying and separating TD from pre-terminal (or normal) decline in physical function is furthermore important as impending mortality may otherwise lead to an overestimation of normative age-related declines (2,28). Finally, a better understanding of TD could also help clinicians to tailor care programs to patient’s needs, and assist in end-of-life decision making (12,29). With the current paper, we aim to provide new direct evidence on whether and when there is a TD in physical function by analyzing extensive longitudinal physical performance data from a long-standing U.S. cohort study with near-complete mortality.

## Method

### Data

We used data from the Yale Precipitating Events Project (PEP) Study (30,31), a cohort study that has conducted comprehensive face-to-face assessments, including physical performance tests, at 18-month intervals in a sample of 754 initially nondisabled health plan members 70 years or older in south central Connecticut (United States) since 1998/1999. The study protocol was approved by the Yale Human Investigation Committee, and all participants provided verbal informed consent. For this study, we used data from 702 (= 93%) participants who died before July 2019.

### Variables

Overall physical function was measured using the Short Physical Performance Battery (SPPB) (32), a well-established instrument with good reliability, validity, and responsiveness (33). SPPB is a combined measure of lower extremity function, strength, and balance. In the PEP study, a modified version of the SPPB is used which combines the standard hierarchical test of balance (0–4) (32) with rapid gait speed (timed 20-foot/6-m walk with a turn in seconds) and the time required to perform 3 chair rises instead of 5 (in seconds). Assignment of 0–4 scores for the gait speed and chair rise test was based on cutpoints from quartiles derived from the first 356 enrolled participants of the PEP Study who had been selected randomly from the source population (30). Finally, a composite SPPB score (range 0–12) was derived as per convention (34) by summing up the scores of the 3 component tasks. In addition to the summary score of the SPPB, we also used the continuous and more fine-grained gait speed and chair rise test scores (in seconds) as secondary outcome measures. For the continuous outcomes, we excluded observations when participants were unable to perform the tasks successfully (12% for gait speed, 20% for the chair rise test). To allow comparison with the more established phenomenon of cognitive TD, we also included global cognitive functioning assessed with the Mini-Mental State Examination (MMSE, range = 0–30) (35).

Additional variables used in this study included time-to-death and age at death (both in years), sex (male/female), years of education, birth cohort (born <1920/1920+), the number of chronic diseases at baseline (0–9), baseline obesity (body mass index [BMI] ≤ 30/BMI > 30), baseline physical activity (Physical Activity Scale for the Elderly [PASE] (36): range = 0–370), and the immediate or underlying condition leading to death. The condition leading to death was classified according to a previous protocol (37) based on death certificates and/or information from the last comprehensive assessment before death as either (1) cancer, (2) organ failure, (3) advanced dementia, (4) frailty, (5) sudden, or (6) other. For more details on how the cause of death was operationalized, see Supplementary Methods 1.

### Statistical Analysis

To assess whether decline in physical performance accelerates in the last years before death, we compared the fit (Watanabe Akaike Information Criterion and *R*-squared) of 3 different generalized mixed regression models: one with a linear trajectory of time-to-death depicting a steady decline without acceleration, one with an added quadratic term depicting a smooth acceleration in decline, and a change point model (22,38) which estimates 2 linear slopes as well as the change point between slopes. The last model, which represents the state-of-the-art modeling approach in TD research (13), most closely resembles the TD hypothesis with the first slope depicting the pre-TD, the second slope the TD, and the change point of the timing of the onset of TD. Importantly, in contrast to the model with the quadratic term, the change point model permits the estimation of the onset of accelerated decline. All 3 models included a random intercept and all respective slope terms for time-to-death (linear; linear + quadratic; linear slope 1 + linear slope 2 + change point), but we restricted correlations between random effects to zero because the change point models would not otherwise converge. To isolate within-person TD from between-person differences with regard to age and time-to-death at baseline (39), we adjusted all fixed parameters for age at death (age at death = age at baseline + time-to-death) (40). Because SPPB test scores are discrete measures (strictly positive integers with a defined upper bound), we used the beta-binomial distribution with a logit link function, that is, modeling the achieved score (0–12) as a count out of total possible counts (12). The same approach was used for the MMSE (41). For the time (in seconds) needed to complete the gait speed test, we used the log-normal distribution, whereas, for chair rise test scores, we used the normal distribution. Selection of appropriate response distributions was based on posterior predictive checks. The above-described mixed-model approach allowed us to estimate not only average trajectories but also to investigate the individual-level variation around average parameter estimates (22,23,40), and to include all available observations, even those from participants who had only 1 assessment of physical performance under the assumption that missingness depends on the observed data (42).

Finally, we assessed between-person differences in the onset of TD using a 2-step procedure (43). First, we extracted change point estimates from the above-described random change point models for all 3 outcomes. Second, we regressed the estimated individual change points on sex, years of education, birth cohort, baseline physical activity, obesity, number of chronic diseases, and cause of death in a multivariate linear regression model.

A more complete description of the statistical models is provided in Supplementary Methods 2.

## Results

As shown in Table 1, about 64% of the participants were women, the mean number of years of schooling was 12.0 (standard deviation [*SD*] = 2.9), the average baseline age was 78.8 years (*SD* = 5.2), and the average age at death was 86.8 (*SD* = 6.3) years. The most common condition leading to death was frailty (26.9%) followed by organ failure (21.4%), advanced dementia (21.1%), and cancer (16.2%). Sudden deaths were rare (2.1%) and the remaining other deaths (12.3%) were mostly due to cardiovascular, infectious, and respiratory diseases. In total, the 702 deceased participants provided 4 133 repeated assessments of SPPB (5.9 measurements per person on average), 3 645 repeated measurements of gait speed (5.2 observations per person on average), and 3 327 repeated measurements of the chair rise test (4.7 observations per person on average). The average length of follow-up before death was 9.1 (interquartile range [IQR] = 8.0, range = 1–20.3) years. The median time (in years) between the last available measurement and death was 1.0 (IQR = 0.9) for the SPPB, 1.5 (IQR = 2.1) for gait speed, and 1.8 (IQR = 2.6) for the chair rise test. 51.1% of the participants had a valid SPPB score during their last year of life, whereas valid continuous measurements of both gait speed and chair rises were available for 22.9% during their last year. More details on the descriptive characteristics of physical function throughout follow-up are shown in Table 2.

**Table 1.** Baseline Characteristics of Study Participants in Total and by Age at Death

Characteristics	Total	Age at Death		
		<80 Years	80–89 Years	90+ Years
Female, *n* (%)	449 (63.9)	59 (60.2)	249 (63.0)	141 (67.5)
Age, mean ± *SD*	78.8 (5.2)	73.8 (2.3)	78.0 (4.5)	82.5 (5.0)
Education (years), mean ± *SD*	11.9 (2.9)	12.2 (2.8)	12.0 (2.8)	11.7 (3.0)
Birth cohort (<1920), *n* (%)	321 (45.7)	3 (3.1)	164 (41.5)	154 (73.3)
Time-to-death, mean ± *SD*	9.2 (5.2)	4.6 (3.5)	8.6 (4.6)	12.4 (4.8)
Chronic conditions, mean ± *SD*	1.8 (1.2)	2.2 (1.3)	1.8 (1.2)	1.5 (1.1)
Obesity (BMI > 30), *n* (%)	152 (21.7)	22 (22.4)	93 (23.5)	37 (17.7)
Physical activity (PASE), mean ± *SD*	86.5 (56.3)	92.3 (66.5)	85.5 (55.5)	85.7 (52.4)
Cause of death				
Cancer, *n* (%)	114 (16.2)	24 (24.5)	72 (18.2)	18 (8.6)
Organ failure, *n* (%)	150 (21.4)	23 (23.5)	82 (20.8)	45 (21.5)
Advanced dementia, *n* (%)	148 (21.1)	6 (6.1)	72 (18.2)	70 (33.5)
Frailty, *n* (%)	189 (26.9)	19 (19.4)	104 (26.3)	66 (31.6)
Sudden, *n* (%)	15 (2.1)	3 (3.1)	11 (2.8)	1 (0.5)
Other, *n* (%)	86 (12.3)	23 (23.5)	54 (13.7)	9 (4.3)

*Notes*: BMI = body mass index; PASE = Physical Activity Scale for the Elderly; *SD* = standard deviation.

**Table 2:** Descriptive statistics of physical performance measures.

Wave	Time	TTD	SPPB	GAIT	CHAIR
	Years	Median (IQR)	Median (IQR)	Median (IQR)	Median (IQR)
1	0.0	9.1 (8.0)	7.0 (5.0)	9.4 (4.7)	9.9 (4.3)
2	1.5	7.9 (7.6)	6.0 (5.0)	9.6 (4.7)	9.6 (4.5)
3	3.0	7.2 (6.9)	6.0 (5.0)	9.6 (5.8)	10.0 (4.4)
4	4.5	6.2 (6.2)	6.0 (5.0)	10.1 (6.4)	10.7 (4.7)
5	6.0	5.5 (5.6)	5.0 (4.0)	10.6 (5.7)	10.5 (4.9)
6	7.5	4.8 (5.2)	5.0 (5.0)	10.5 (7.0)	10.4 (4.6)
7	9.0	3.9 (4.8)	4.0 (5.0)	11.0 (7.1)	10.6 (5.0)
8	12.0	3.2 (3.9)	2.0 (3.0)	13.1 (8.5)	11.4 (4.4)
9	13.5	2.7 (3.1)	3.0 (3.0)	13.8 (10.4)	13.87 (4.6)
10	15.0	2.4 (2.8)	2.0 (4.0)	13.7 (8.5)	14.2 (6.1)
11	16.5	1.4 (2.0)	3.0 (3.0)	17.7 (8.7)	15.0 (6.2)
12	18.0	1.5 (1.0)	2.0 (3.0)	20.7 (14.4)	17.5 (13.6)
13	19.5	0.6 (0.4)	0.0 (1.0)	36.8 (30.6)	21.8 (14.3)

*Notes*: No comprehensive assessment was conducted at 10.5 years of follow-up due to lack of funding. CHAIR = chair rise test score in seconds, GAIT = rapid gait speed in seconds, IQR = interquartile range; SPPB = Short Physical Performance Battery, TTD = time-to-death.

**Figure 1.**
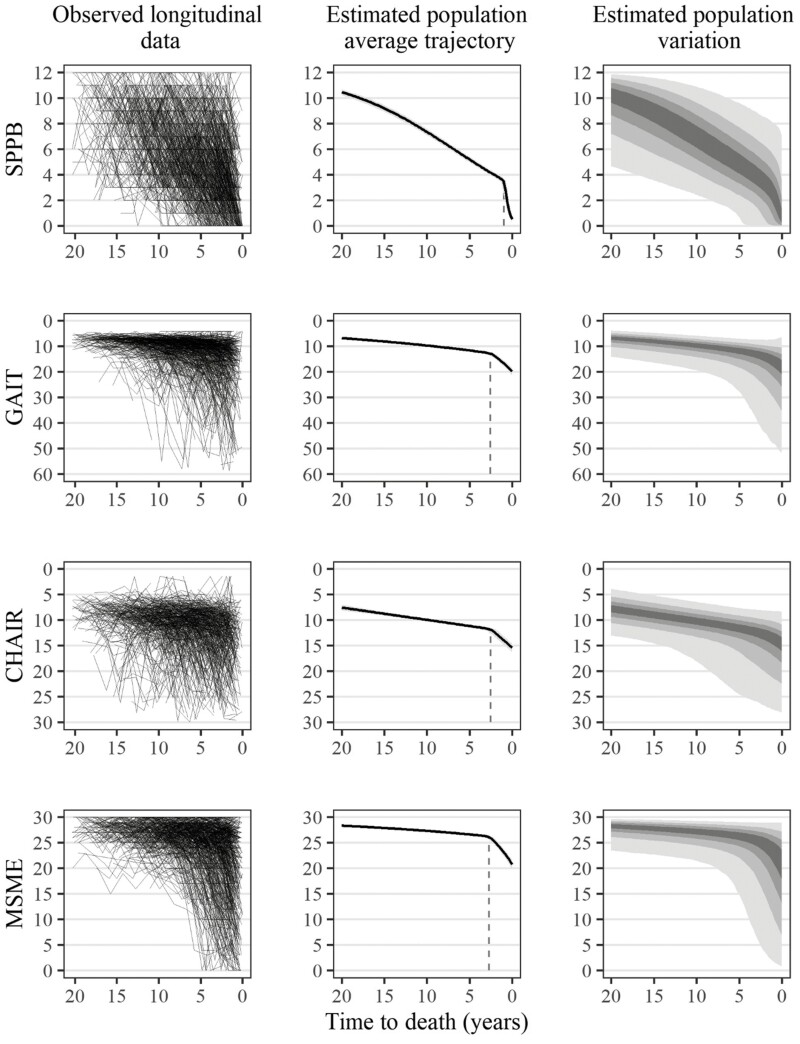
Observed physical performance scores, estimated average trajectories, and estimated trajectory variation. Sample size = 702 participants and 4 133 (SPPB), 3 645 (gait speed test), 3 327 (chair rise test), and 4 ,133 (MMSE) observations, respectively. Lines in spaghetti plots (first column) refer to observed individual trajectories. Population-average trajectories (second column) are based on mean posterior predictions of fixed effects and adjusted for age to death, light gray shading refers to 95% credible interval. Estimated population variation (third column) is based on posterior predictions for a not yet observed older person using both fixed and random effect variance and shows prediction bands of 25% (dark gray), 50%, 75%, and 95% (light gray). CHAIR = chair time score in seconds; GAIT = rapid gait speed in seconds; SPPB = Short Physical Performance Battery; MMSE = Mini-Mental State Examination.

Model fit comparison (Supplementary Table 1) showed that for all 3 outcomes of physical function, the change point models described the data better than the linear or quadratic time-to-death trajectories. The main findings of this analysis, that is, the random change point models, are shown in Table 3 and visualized in Figure 1. In Figure 1, for overall physical function (SPPB), gait speed and chair rise test scores as well as cognitive functioning (in rows), we first show the raw individual-level longitudinal observations (first column), the estimated population-average trajectory according to time-to-death which includes pre-terminal and terminal slopes (second column), and the estimated population-level variability around the average trajectories (third column). The most clear-cut pattern of TD is visible for cognitive functioning: A pre-terminal phase with limited average decline (0.13 points per year) for most of the period under study was followed by a steep TD (2 points per year), that is, a 15-times steeper decline. TD in cognition began on average 2.7 years (95% credible interval [CI] = 2.2, 3.2) before death. The onset of TD in cognition varied across individuals by 0.5 years (= random-effect variance, expressed as ±1 *SD*).

**Table 3.** Estimated Within-Person Trajectories: Results From Random Change Point Models

	SPPB	GAIT	CHAIR	MMSE
	Coef (95% CI)	Coef (95% CI)	Coef (95% CI)	Coef (95% CI)
Fixed effects				
Intercept (β_10_)	−0.86 (−0.97, −0.75)	2.52 (2.45, 2.57)	11.78 (11.31, 12.22)	1.94 (1.86, 2.03)
Age at death (β_11_)	−0.06 (−0.07, −0.05)	0.02 (0.01, 0.02)	0.15 (0.10, 0.20)	−0.03 (−0.05, −0.02)
Pre-terminal slope (β_20_)	0.15 (0.14, 0.16)	−0.04 (−0.04, −0.03)	−0.24 (−0.28, −0.20)	0.05 (0.04, 0.06)
Age at death (β_21_)	0.00 (0.00, 0.00)	0.00 (0.00, 0.00)	−0.01 (−0.02, 0.01)	0.00 (0.00, 0.00)
Terminal slope (β_30_)	2.46 (1.50, 43.88)	−0.18 (−0.22, −0.14)	−1.47 (−1.82, −1.18)	0.41 (0.35, 0.49)
Age at death (β_31_)	0.01 (−0.10, 0.12)	0.00 (0.00, 0.01)	0.02 (−0.02, 0.07)	0.00 (−0.01, 0.01)
Change point (ω_0_)	−3.02 (−3.40, −2.64)	−1.95 (−2.29, −1.59)	−1.98 (−2.39, −1.60)	−1.85 (−2.08, −1.65)
Age at death (ω_01_)	0.06 (0.03, 0.09)	0.06 (0.03, 0.08)	0.06 (0.02, 0.10)	0.05 (0.03, 0.08)

Notes: *N* = 702 participants. Fixed-effect coefficients are on the log odds scale except for chair rise test. Numbers in parentheses are 95% credible intervals (95% CIs). CHAIR = time required to perform chair stands in seconds; GAIT = rapid gait speed in seconds; MMSE = Mini-Mental State Examination; *SD* = standard deviation SPPB = Short Physical Performance Battery. Models fit with Hamiltonian Monte Carlo (HMC) sampling procedure with 4 chains and 2 000 post-warmup iterations per chain. All $\hat{r}\text{-}values1.05.$

The average trajectory of SPPB also showed 2 distinct slopes, and a late change point 1.0 (95% CI = 0.7, 1.4) year before death, which also varied across individuals (±1 *SD* = 0.7 years). In contrast to the MMSE, and the continuous scores from its two subcomponents, SPPB scores showed a considerable decline (0.4 points per year) across most of its range before TD set in. Nonetheless, the TD in SPPB during the very last year of life amounted to 2.9 points (per year), which makes it still 8 times steeper compared to the pre-terminal phase. The overall decline pattern in the more fine-grained chair rise and gait speed test scores resembled the MMSE pattern rather than SPPB (Figure 1). For the chair rise test, the pre-terminal slope was 0.3 seconds per year compared to 1.4 in the terminal phase (6 times steeper), which began, on average 2.5 (95% CI = 1.7, 3.4; ±1 *SD* = 2.6) years before death. For gait speed, the time to complete the test increased by 0.4 seconds per year during the pre-terminal phase, whereas in the TD phase, which commenced 2.6 (95% CI = 1.8, 3.5; ±1 *SD* = 1.6) years before death on average, this increased to 2.7 seconds per year (8 times steeper). The prediction bands (25%, 50%, 75%, and 95%) around the average trajectory (third column) imply that there is a considerable but stable level of heterogeneity in SPPB between participants. In contrast, there were clear increases in heterogeneity during the last 5 years of life, and particularly during the TD phase, in the 2 continuous physical performance measures, as well as in the MMSE. For most participants, the onset of TD according to gait speed (71%) and chair rise (70%) test scores occurred later than the onset of cognitive TD, on average by 3 months. The estimated onset of TD in the summary measure of overall physical function (SPPB) was closer to death for 96% of participants compared to gait speed, and 89% compared to chair rise test scores (Supplementary Figure 2). In summary, both gait speed and chair rise measured in seconds are earlier markers of TD in physical function than SPPB. Age at death, which we included to isolate within-person changes from between-person differences in chronological age and time-to-death, moderated within-person TD in physical function (Supplementary Figure 1): Older adults who died earlier had higher levels of functioning at the change point as well as a later onset of TD compared to those who died later.

In the next step, we extracted the estimated individual-level change points for further inspection. We found that the timing of the onset of TD correlated between SPPB and global cognition (bivariate Pearson correlation coefficient *r* = 0.48, 95% CI = 0.42, 0.54) as well as between gait speed and chair rise test scores (*r* = 0.52, 95% CI = 0.47, 0.58), but associations were small (*r* < 0.35) otherwise. Finally, we modeled between-person differences in the onset of TD in physical function using the estimated individual change points from the first set of models as new outcome variables. Sociodemographic and health-related predictors were able to explain a considerable amount of variance (SPPB: 18%, gait speed: 14%, chair rise test: 18%) in the estimated change points between participants (Table 4). Older adults born earlier (<1920) had between 1 (gait speed) and 4 (chair rise) months later change points compared to those born later (1920+). The number of chronic diseases (at baseline) was not associated with the onset of TD. Participants who were very physically active (at baseline) entered TD 3 months (chair rise) later, and those who were obese 2 months (gait speed, chair rise) earlier compared to inactive respectively nonobese participants. Differences also showed by cause of death: participants who died from dementia entered TD earlier (SPPB = 6 months, gait speed = 3 months, chair rises = 1 month) compared to those whose underlying condition leading to death was frailty. Cancer deaths, in contrast, had a considerably later onset of TD: SPPB = 3 months, gait speed = 3 months, chair rises = 5 months. Participants with sudden death or other causes of death resembled cancer deaths in their TD trajectory. The residual correlation coefficients from the multivariate model still imply a moderate association between the onset of TD in gait speed and chair rises, and lower correlations between SPPB and its continuous subdomains.

**Table 4.** Between-Person Differences in the Onset of Terminal Decline In Physical Function: Results from the Multivariate Regression Model

	SPPB	GAIT	CHAIR
	Coef (95% CI)	Coef (95% CI)	Coef (95% CI)
Female (β_1_)	−0.01 (−0.12, 0.11)	0.08 (0.01, 0.15)	0.12 (0.03, 0.27)
Years of education (β_2_)	0.00 (−0.01, 0.02)	0.00 (−0.01, 0.01)	−0.01 (−0.03, 0.02)
Born <1920 (β_3_)	−0.19 (−0.31, −0.08)	−0.10 (−0.17, −0.03)	−0.31 (−0.46, −0.17)
Chronic diseases (β_4_)	0.04 (−0.01, 0.09)	0.00 (−0.03, 0.02)	−0.01 (−0.07, 0.05)
Obesity (β_5_)	0.12 (−0.01, 0.25)	0.15 (0.07, 0.23)	0.17 (0.00, 0.34)
Physical activity (2 *SD*) (β_6_)	−0.05 (−0.16, 0.07)	−0.08 (−0.15, −0.01)	−0.28 (−0.44, −0.13)
COD: dementia (β_7_)	0.54 (0.39, 0.70)	0.17 (0.08, 0.27)	0.12 (−0.09, 0.32)
COD: organ failure (β_8_)	−0.01 (−0.16, 0.15)	0.00 (−0.09, 0.09)	−0.18 (−0.39, 0.01)
COD: cancer (β_9_)	−0.28 (−0.44, −0.11)	−0.21 (−0.31, −0.11)	−0.42 (−0.64, −0.20)
COD: sudden death (β_10_)	−0.20 (−0.58, 0.17)	−0.27 (−0.49, −0.04)	−0.63 (−1.11, −0.16)
COD: other/unknown (β_11_)	−0.25 (−0.43, −0.07)	−0.27 (−0.49, −0.04)	−0.52 (−0.77, −0.27)
SPPB × CHAIR (COR)	0.21 (0.13, 0.28)	—	0.21 (0.13, 0.28)
SPPB × GAIT (COR)	0.23 (0.16, 0.30)	0.23 (0.16, 0.30)	—
GAIT × CHAIR (COR)	—	0.45 (0.39, 0.51)	0.45 (0.39, 0.51)
*R*-squared	0.18 (0.12, 0.22)	0.14 (0.10, 0.18)	0.18 (0.13, 0.22)

*Notes*: *N* = 673 participants, 29 participants had missing data in 1 or more of the predictor variables. Negative coefficients refer to change points later in life, positive values refer to earlier change points (in years). Physical activity was divided by 2 standard deviations (2 *SD*); therefore, the coefficient can be interpreted as the difference between very low and very high levels of physical activity. Numbers in parentheses are 95% credible intervals (CIs). CHAIR = chair rise test; COD = cause of death; COR = residual Pearson correlation coefficient (after adjustment for predictors); SPPB = Short Physical Performance Battery. Models fit with Hamiltonian Monte Carlo (HMC) sampling procedure with 4 chains and 2 000 post-warmup iterations per chain. All *r*ˆ-values <1.05.

## Discussion

In this study, we examined whether and when physical function based on objective performance measures follows a TD trajectory, that is, whether a long-term age-related minor decrease in functioning is followed by a steep, nonlinear within-person decline of function during the very last years of life. We found evidence that supports the TD hypothesis: The decline in both the overall measure of physical function (SPPB) and the more fine-grained gait speed and chair rise test scores accelerated substantially (six to eightfold) in the very last year(s) of life. TD in physical function followed TD in cognitive functioning time-wise and showed a similar overall pattern in both gait speed and chair rise test scores. Heterogeneity in functioning increased in gait speed and chair rise test scores after the TD phase set in. There were differences in the onset of TD by cause of death: The TD phase began earliest in older adults who died from dementia and the latest in those who died from cancer, leading to differences of 6–9 months.

Research on TD spans decades and has, from its early beginnings (10,11) to recent summaries provided by systematic reviews (12,13), focused mostly on late-life cognition. In contrast, research on whether physical function also exhibits a terminal phase of steep decline in the last years before death—akin to cognition—has remained sparse. There is, however, indirect evidence that links declines in physical function to mortality risk (2,14–17), and direct evidence from a single study that documents TD in physical function based on data from the Religious Orders Study (23). Our results from the PEP study based on a highly similar statistical modeling approach but a different sampling frame (drawn from the general older population), follow-up lengths and intervals, as well as the measurement of gait speed and global cognition, match those from Wilson and colleagues closely: In both studies, the average onset of TD in global cognition was estimated at 2.7 and 2.8 years before death, respectively, which was followed within a few months later by the onset of TD in gait speed. Further similarities between the 2 studies are that the steepness of TD compared to the pre-terminal rate of decline was 2–3 times higher in global cognitive function compared to global physical function, and that there was a considerable amount of heterogeneity across participants when TD in both cognitive and physical function set in. Similar to the study of Wilson et al. (23), we also found that those older adults who died at a higher age tended to have a lower level of functioning as they entered the TD phase. We, however, found no difference in the rate of TD according to age at death. Instead, the onset of TD increased with age at death in our study, resulting in a more drawn-out TD phase for those who died later in life.

There are several more notable differences between our study and the work of Wilson and colleagues (23). Compared to their fine-grained but ad-hoc created composite measure of physical function, we instead used the established and validated (33) SPPB instrument (32) as overall measure of physical function. The SPPB did not only show considerable and meaningful (44) decline before the change point—which is at odds with the notion of the pre-terminal phase—but also yielded a change point estimate considerably closer to death. This difference between studies, as well as the difference between the SPPB and its 2 continuous subcomponents within our study could be due to the coarse nature of the SPPB which relies on categorized subcomponents. Indeed, there was clear evidence for a floor effect from the sixth wave onward, where more than 20% of the participants achieved the lowest possible SPPB score. Relatedly, this could also be because the SPPB is less affected by censoring of measurements in proximity close to death. Even participants who could not successfully complete 1 or more of the subtests, for example, due to severe cognitive or physical impairment, obtained a valid (low) score in the SPPB, whereas such observations are classified as missing in the continuous test scores and were implicitly imputed during estimation based on the observed data (42). Unfortunately, we cannot untie the trade-off between more fine-grained measurements on the one hand (gait speed and chair rise tests in seconds) and more measurements closer to death (SPPB) on the other. However, because early detection of (terminal) declines in physical function is important for potential medical interventions aimed at slowing further deterioration and for care planning and end-of-life decision making (12,29), single fine-grained continuous measures of physical function might be preferable over the coarser SPPB. Indeed, the gait speed and chair rise test, both of which identified the onset of TD more than 2 years before death in our study, have been aptly described as multisystem performance tests that can effectively track older adults’ overall health status (3). Similarly, a recent study (24), based on the data from the U.S. Health and Retirement Study, showed that increases in the frailty index (45), also a comprehensive but more fine-grained measure of older adults’ overall health, relative to the SPPB, accelerated markedly (fivefold) during the last 3 years of life. Collectively, these results suggest that the TD phenomenon is not restricted to the cognitive domain but extends to physical health, which is not surprising given the strong associations between cognition and physical functioning (46).

Although knowledge about the onset and shape of TD in physical function is important to advancing our understanding of end-of-life processes, the clinical relevance of the average onset of TD for gait speed and chair rise test scores might be questioned. First, our results indicate that for all 3 outcomes of physical function, there is considerable heterogeneity between older adults when TD sets in and how fast older adults’ health subsequently deteriorates, which, in the case of gait speed and chair rise test scores, further increased substantially during the very last years of life. This heterogeneity should be kept in mind when thinking about whether a presenting individual older adult might or might not have already entered the TD phase. Second, the analysis of TD is—by necessity—a retrospective analysis of trajectories of deceased older adults and does not permit prospective prediction in either populations or individuals. Whether “terminal-like declines” (8)—that is, declines in physical function that resemble a TD but may or may not result in death—have translational value for individual-level prognosis has not yet been shown. Although previous research indicates that declines in physical functioning can, in general, predict mortality (2,14–17), these studies are often not designed to detect sharp, short-term, nonlinear declines, and the predictive value of these declines tends to greatly diminish after adjustment for current health status or level of functioning (17,47,48). A recent study (49) among a sample of Dutch oldest old who were assessed at 9-month intervals, indicated, in contrast, that increases in the frailty index during the previous year were indeed more important than the current frailty level, which could reflect a predictive potential of “terminal-like declines.”

Our study also assessed between-person differences in the within-person onset of TD. Using a 2-step procedure, we found that participants who were born later and those who were physically inactive or obese at baseline experienced an earlier onset of TD, particularly in the chair rise test. We are not aware of any prior studies that have evaluated obesity- or physical activity with regard to TD, although they have been identified as risk factors for mobility loss in later life more generally (3). With regard to the effect of birth cohort, a study by Hülür et al. (50) reported that the later-born cohort showed a steeper cognitive TD than the earlier-born cohort, which contrasts with our finding that the later cohort had an earlier onset of TD in physical function, that is, the physical TD phase tends to be a somewhat more drawn-out process for those born later. Research explicitly investigating TD rarely considers the underlying or immediate condition leading to death as a potentially important contributor to between-person differences (12), although its importance has been well recognized in a separate strand of end-of-life-related research that focuses on the very last year or even months of life. Work by Lunney and colleagues (26), and more recently by Stolz et al. (27), for example, suggests that disability trajectories vary considerably by cause of death: Those with cancer tend to be highly functional until they experience a sharp decline during the last months of their life, whereas those with dementia or frailty have higher overall levels of self-reported disability and less clear-cut TD phases. In the current study, we also found the latest onset of TD in objectively measured physical function in older adults who died from cancer, and a more drawn-out TD phase among those who died from dementia. Wilson et al. (23) reported that those with neuropathologic measures (plaques and tangles) had an earlier onset of TD in motor function, which is compatible with our findings that older adults who died from dementia experienced an earlier onset of TD in physical function, and therefore also a longer end-of-life period characterized by a low level of functioning. An earlier onset of and hence longer TD phase with low(er) levels of functioning would likely lead to an earlier and greater demand for assistive devices and long-term care services.

The above-discussed between-person differences in how TD unfolds during the last years of life raise the question as to what mechanism(s) may drive TD in physical function. Ferrucci et al. (3) have referred to reductions in functional reserves due to aging-related changes in many physiological systems. In the setting of an acute health event such as a fall injury or illness leading to hospitalization or the progression of a chronic health problem such as cardiovascular or respiratory disease, these reductions increase the likelihood of a visible and steep (terminal) decline of physical function in late life. Although our understanding of the specific mechanisms of TD is limited (9), Wilson et al. (23) speculated that damage in the brain could impair both cognitive and subsequently physical functioning, and they argued that the broader array of physiologic systems that underlie physical function—including the spinal cord, the peripheral nervous system and muscle—are subject to multiple processes, for example, immune, inflammatory, endocrine, metabolic, which may be responsible for TD. One potential explanation is that several of these processes may lead to impaired organ function, which, in turn, adversely affects neuromuscular function. However, as of yet, there are no empirical studies linking differences or changes in biomarker data to declines in physical performance measures in late life. Similarly, Landré and colleagues (21) suggested that disease- and age-related loss of muscle mass along with chronic diseases might contribute to declines in physical function preceding death. Both the study by Landré and ours, however, found little association between the number of chronic diseases and TD.

The current study is, to the best of our knowledge, only the second to rigorously examine TD in physical function. Specifically, based on extensive longitudinal data from a cohort with near-complete mortality, we were able to statistically separate within-person pre-terminal from TDs and explicitly estimate the onset of TD using random change point models. Further strengths of the current study include the use of multiple established and validated objective performance measures of physical function, the comparison with cognitive TD, as well as the overall high data quality, that is, the long follow-up time (20 years) in a sample from the general older population, the relatively high number of interviews per person (5–6 on average), and the availability of measurements in proximity to death (last measurement 1–2 years before death on average). However, several notable limitations warrant comment. First, in this longitudinal study, we could establish associations, but not causality, that is, whether the onset of TD in physical function can be actually postponed by health-related behavior. Second, because study participants were members of a single regional health plan in a small urban area and were initially nondisabled, our results might not be generalizable to older adults in other settings. However, the demographics (age, sex, and education) of our cohort are largely comparable to those of older adults in greater New Haven (CT) and to those of the general U.S. population except for the under-representation of non-White older adults. The generalizability of our results is further supported by the high participation rate (75%) and the low attrition rate (between 4% and 11% refused participation in comprehensive assessments) (31), as well as the near-complete mortality (93%) in the PEP study. Third, we were not able to freely estimate the correlation coefficients between the random effects to assess how the pre-terminal slope, the terminal slope, the level of functioning at the change point, and the timing of the change point were related to each other at the individual level, as the model would not converge upon adding 6 additional random-effect parameters. Fourth, our study assessed trajectories of physical function in the last years of life *after* participants had died. Future studies should aim to provide more translational value by assessing whether “terminal-like”—that is, steep, nonlinear—declines in physical function in individuals can dynamically predict short-term mortality using, for example, the flexible framework of joint models for longitudinal and time-to-event data.

To conclude, based on extensive longitudinal physical performance data from a cohort with near-complete mortality analyzed with random change point models, the current study provides direct evidence that there is TD in physical functioning. TD began on average about 2 and a half years before death based on continuous gait speed and chair rise test scores and 1 year before death according to the coarser but less-censored SPPB. We found evidence for between-person differences in the onset of TD according to age at death, birth cohort, health-related behavior, and cause of death. Our findings confirm those from an earlier study (23) suggesting that a considerable part of late-life decline in physical function is mortality related, that is, it may be due to the underlying physiological impairments that also lead to death.

## Supplementary Material

glad119_suppl_Supplementary_Material

## Data Availability

Requests for access to data from the PEP Study for meritorious analyses from qualified investigators should be directed to Thomas Gill (thomas.gill@yale.edu). The R-Markdown code reproducing all analyses, results and this manuscript are available online (https://osf.io/au5vh/).
