# Supplementary material for: Terminal Decline in Physical Function in Older Adults
Source: J Gerontol A Biol Sci Med Sci. 2023 May 6;79(1):glad119. doi: 10.1093/gerona/glad119 (PMC10733182; doi:10.1093/gerona/glad119)
Supplement: glad119_suppl_Supplementary_Material [file glad119_suppl_supplementary_material.docx]

**Supplementary Methods 1: The operationalization of cause of death**

Cancer deaths were classified based on the following codes of the International Classification of Diseases 10th revision (ICD-10) from death certificates: C00.0-C97, D37.0-D37.9, D44.0-D48.7.

Organ failure was also based on death certificates and included congestive heart failure (ICD-10: I25.5, I42.0-I42.9, I50.0-I51.9), chronic lung disease (ICD-10: J43.0, J44.9, J47, J61, J84.0-J84.9), chronic kidney disease (ICD-10: N18.0-N18.9), and liver cirrhosis (ICD-10: K74.0-K74.6).

Severe dementia was based on either death certificates (ICD-10: F01.0-F01.9, F03, G30.0-G30.9, R54, A81.0) or a MMSE score of less than 10 in the last comprehensive assessment.

Phenotypic frailty was considered when three or more of the five following criteria from the last comprehensive assessment applied: weight loss, exhaustion, low physical activity, muscle weakness, slow gait speed. Weight loss was defined as a positive answer to the question "In the past year, have you lost more than 10 pounds?". The exhaustion criterion was met if participants answered "Much or most of the time" to one or both of the following two statements from the Center for Epidemiologic Studies Depression Scale: "I felt that everything I did was an effort" and "I could not get going". Low physical activity applied to men/women who scored >64/<52 points on the Physical Activity Scale for the Elderly. Muscle weakness was defined as grip strength in the lowest 20% (adjusted for sex and self-reported body mass index) measured by a handheld dynamometer. Finally, slow gait speed was defined as requiring more than 10 seconds in the rapid gait test during the last comprehensive assessment.

Sudden death was defined as not meeting the criteria for cancer, advanced dementia, frailty, organ failure, and not having a history of cancer, heart disease, chronic lung disease, hip fracture or stroke, and not living in nursing home at time of death.

Other causes of death did not meet criteria for cancer, advanced dementia, frailty, organ failure, or sudden deaths. Other causes of death included mostly (70%) diseases of the circulatory system (except congestive heart failure) but also infectious or respiratory diseases (21%) as the immediate or underlying cause of death.

When there were multiple conditions leading to death, assignment to a unique cause of death was forced hierarchically in the following order: cancer, dementia, organ failure, frailty, sudden death.

**Supplementary Methods 2: Statistical models**

The mixed regression model with the linear trajectory of time to death was outlined as:

*y_ij_* =*β*_1i_ + *β*_2i_ + *ϵ_ij_*

*β*_1i_ *_=_ β*_10_ + *β*_1_*AgeDeath_i_* + *u*_1_*_i_*

*β*_2i_ *_=_ β*_20_ + *β*_2_*AgeDeath_i_* + *u*_2_*_i_*

where observed measurements of physical function (*y*) for the *i^th^* person (i = 1, ..., N) at time points *t_ij_* (j = 1, ..., *n_i_*) were modelled as a function of an older adult’s intercept (*β*_1i_) and linear slope (*β*_2i_) as well as the residual error (*ϵ_ij_*). Individual’s intercept and slope consist of the (fixed) population-average intercept (*β*_10_) and slope (*β*_2i_) and the (random) individual-level deviation from these (*u*_1_*_i_* and *u*_2_*_i_* ) as well as the adjustment for age at death (*β*_1_ and *β*_2_).

The model with the quadratic trajectory of time to death was outlined as:

*y_ij_* =*β*_1i_ + *β*_2i_ + *β*_3i_ *+ ϵ_ij_*

*β*_1i_ *_=_ β*_10_ + *β*_1_*AgeDeath_i_* + *u*_1_*_i_*

*β*_2i_ *_=_ β*_20_ + *β*_2_*AgeDeath_i_* + *u*_2_*_i_*

*β*_3i_ *_=_ β*_30_ + *β*_3_*AgeDeath_i_* + *u*_3_*_i_*

which just adds a quadratic term for time-to-death in both fixed (*β*_30_) and random effects (*u*_3_*_i_*).

The random change point model, finally, was outlined as:

*y_ij_* =*β*_1i_ + *β*_2i_(t_ij_-ω_i_)I(t_ij_≤ω_i_) + *β*_3i_(t_ij_-ω_i_)I(t_ij_>ω_i_) *+ ϵ_ij_*

*β*_1i_ *_=_ β*_10_ + *β*_1_*AgeDeath_i_* + *u*_1_*_i_*

*β*_2i_ *_=_ β*_20_ + *β*_2_*AgeDeath_i_* + *u*_2_*_i_*

*β*_3i_ *_=_ β*_30_ + *β*_3_*AgeDeath_i_* + *u*_3_*_i_*

ω_i =_ ω_0_ + *β*_4_*AgeDeath_i_* + *u*_4_*_i_*

Here, the observed measurements of physical function (*y*) for the *i^th^* person (i = 1, ..., N) at time points *t_ij_* (j = 1, ..., *n_i_*) were modeled as a function of an older adult’s intercept (*β*_1i_) referring to the expected value at the change point, his/her pre-terminal slope (*β*_2i_) before the change point, his/her terminal slope (*β*_3i_) after the change point, and the timing of the fixed change-point (*ω*_i_) itself. Individual’s intercept, pre-terminal slope, terminal slope, and change point consist of the (fixed) population-average effects (*β*_10,_ *β*_20,_ *β*_30,_ ω_0_) and the (random) individual-level deviation from these (*u*_1_*_i_*-*u*_4_*_i_* ) as well as the adjustments for age at death (*β*_1_-*β*_4_).

Finally, to assess between-person differences in the onset of terminal decline, we extracted the estimated individual change points (*ω_i_* = *ω*_0_ *− u*_4_*_i_*) from the mixed regression models as described above, and regressed them on several between-person characteristics in a multivariate linear regression model:

*ω_i_* = *β*_01_*FemaleSex_i_* + *β*_02_*YearsEdu_i_* + *β*_03_*BornAfter*1920*_i_* + *β*_04_*Chronic_i_* + *β*_05_*Obesity_i_* + *β*_06_*PhysAct_i_* +

*β*_07_*COD*:*Dementia_i_* + *β*_08_*COD*:*OrgFailure_i_* + *β*_09_*COD*:*Cancer_i_* + *β*_10_*COD*:Sudden + *β*_11_*COD*:*Other_i_* + *ϵ_i_*

All models were estimated using R (v4.1.2). The generalized mixed regression models with random change points and the multivariate linear regression models were estimated in a Bayesian framework using R-package brms (v2.17), an interface for the Stan programming language (v2.21.2). The posterior distribution was sampled using weakly informative priors (N (0, 2)) for all parameters and Hamiltonian Monte Carlo with four chains and >2,000 postwarm-up samples per chain. All models passed standard diagnostic criteria, i.e. $\hat{r}<1.05$ and bulk and tail effective sample sizes >400. Details of the data preparation and the statistical analyses are documented in the R-Markdown code-file available online: https://osf.io/au5vh/.

**Table S1: Model fit comparison**

|  | SPPB | GAIT | CHAIR |
| --- | --- | --- | --- |
| WAIC |  |  |  |
| Change point model | 15012 (14821, 15204) | 18418 (18027, 18809) | 17060 (16748, 17372) |
| Quadratic | 15903 (15694, 16112) | 19125 (18758, 19492) | 17540 (17268, 17812) |
| Linear | 16037 (15832, 16243) | 19220 (18862, 19578) | 17608 (17340, 17876) |
| Bayesian R-squared |  |  |  |
| Change point model | 0.80 (0.79, 0.80) | 0.70 (0.68, 0.72) | 0.65 (0.63, 0.67) |
| Quadratic | 0.76 (0.75, 0.77) | 0.52 (0.49, 0.54) | 0.55 (0.53, 0.58) |
| Linear | 0.75 (0.74, 0.76) | 0.50 (0.47, 0.52) | 0.54 (0.52, 0.56) |

Model fit comparison between model using for time-to-death either a linear term, a linear + quadratic term, or a change point model approach. Model fit comparison is based on relative fit using the Watanabe Akaike Information Criterion (WAIC) and absolute fit using Bayesian R-squared. Smaller WAIC and larger R-squared values imply better model fit. Numbers in parentheses are 95% credible intervals. SPPB = Short Physical Performance Battery, GAIT = rapid gait speed test, CHAIR = chair rise test.


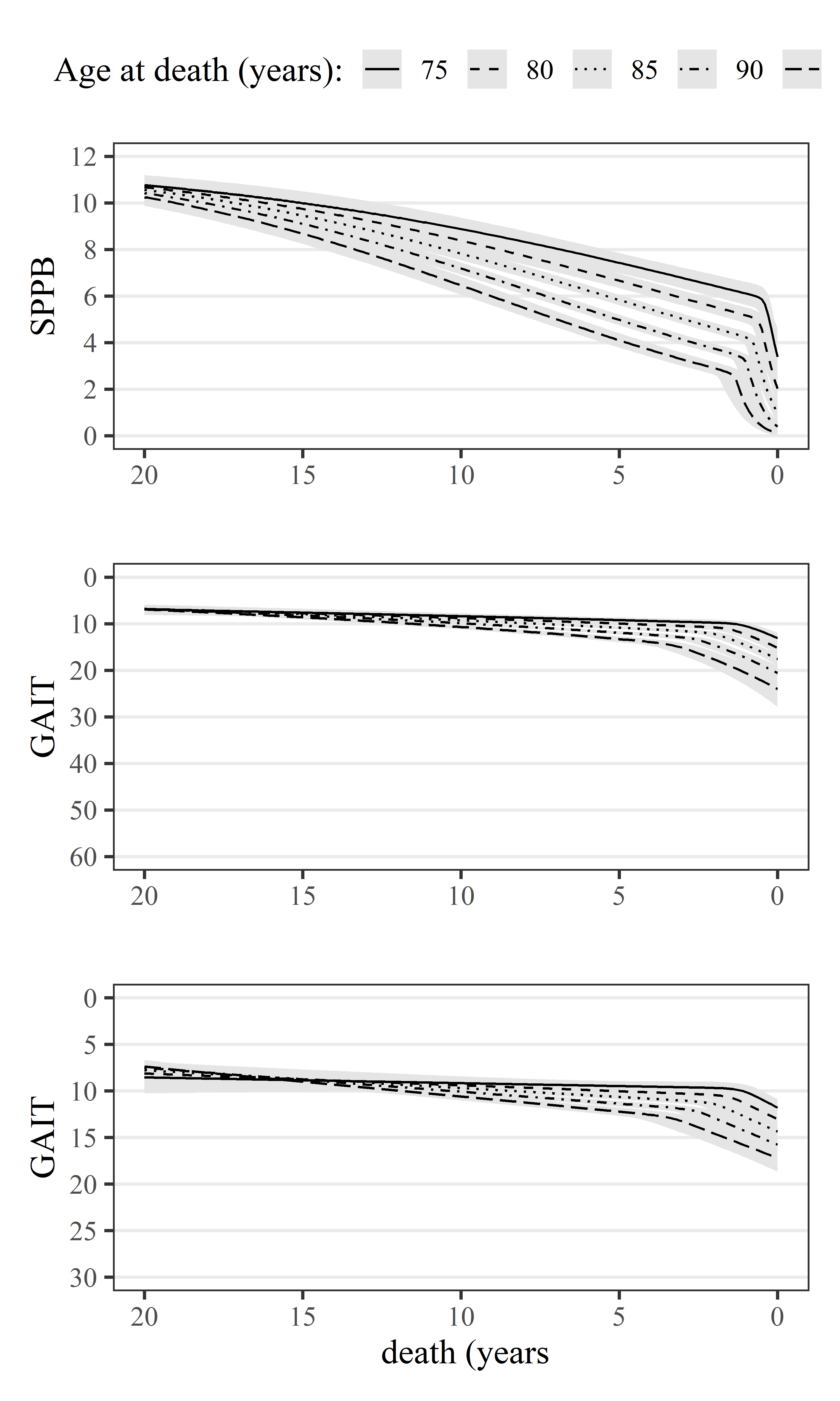
**Figure S1: Estimated average within-person trajectories by age at death**

Trajectories are based on mean posterior predictions of fixed effects only, light grey shading refers to 95% credible interval. SPPB = Short Physical Performance Battery, GAIT = rapid gait speed in seconds, CHAIR = chair time score in seconds

**Figure S2: Distribution of estimated individual change points by outcome**


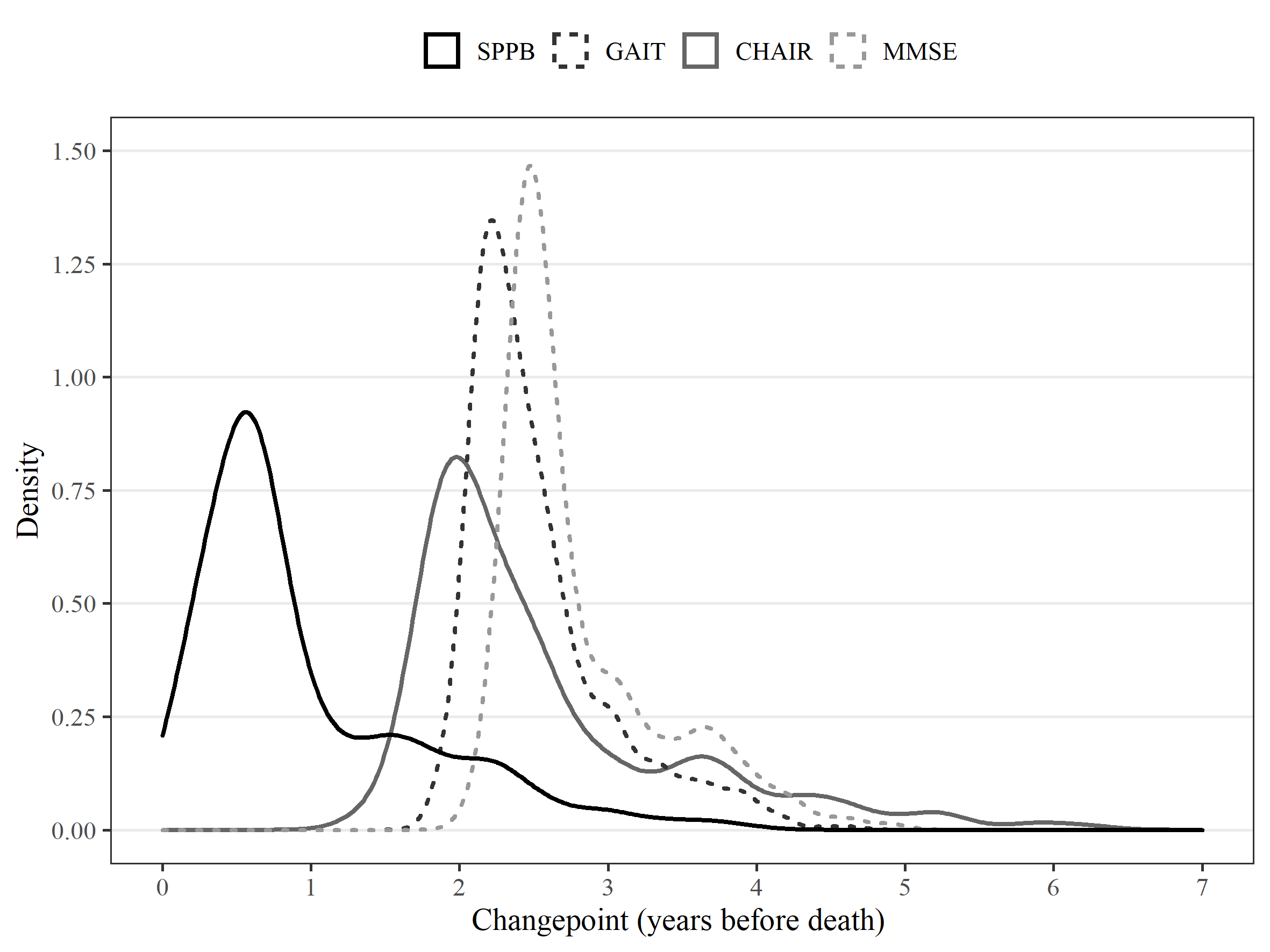


Estimates of individual change points come from mixed models with random change points.
